# The development and implementation of a new import duty on palm oil to reduce non-communicable disease in Fiji

**DOI:** 10.1186/s12992-018-0407-0

**Published:** 2018-08-29

**Authors:** Jeremaia Coriakula, Marj Moodie, Gade Waqa, Catherine Latu, Wendy Snowdon, Colin Bell

**Affiliations:** 1 Pacific Research Centre for the Prevention of Obesity and Non-Communicable Diseases (C-POND), College of Medicine Nursing and Health Sciences, Fiji National University, Private Mail Bag, Tamavua, Suva, Fiji Islands; 2 Deakin Health Economics, Centre for Population Health Research, Faculty of Health, Deakin University, Geelong, Australia; 3 Global Obesity Centre, Centre for Population Health Research, Faculty of Health, Deakin University, Geelong, Australia

**Keywords:** Palm oil, Policy, Non communicable disease, Government

## Abstract

**Background:**

Non communicable diseases (NCD) place a significant health burden on Pacific Island countries including Fiji. Policy interventions to curb NCDs have been implemented in Fiji including a 32% increase in the import duty on palm oil. This study aims to analyse the development and implementation of the increase in palm oil import duty in Fiji. Also, to document the policy process, identify barriers and facilitators during implementation and to examine the impact of the new import duty on import volumes.

**Methods:**

Data were collected through key informant interviews with private stakeholders, government officials and supermarket managers. Transcripts were analysed thematically. Import volumes were analysed for the 2010–2015 period.

**Results:**

Facilitators of policy development and implementation included stakeholder awareness of the health implications of palm oil, preparation of a comprehensive policy briefing paper, and inter-sectoral support and leadership. This decrease in the availability of palm oil was encouraging however, it may have been counteracted to some extent by industry relabelling the product as vegetable oil.

**Conclusions:**

Barriers to policy changes need to be anticipated during the policy development process. Whilst the decline in imports probably reduced population consumption, further research is needed to determine if this translated to a population wide reduction in saturated fat.

**Electronic supplementary material:**

The online version of this article (10.1186/s12992-018-0407-0) contains supplementary material, which is available to authorized users.

## Background

Non-communicable diseases (NCD) are the principal cause of deaths globally resulting in an estimated 38 million deaths in 2012 alone [1]. NCDs account for around 70–75% of all deaths in the Pacific Islands [2]. In Fiji, an estimated 80% of all deaths are attributed to NCDs [3] and poor diets are a major contributing factor.

The Pacific Region is home to seven of the top ten most obese nations in the world [4]. Efforts to prevent and manage obesity in Fiji are ongoing. The Fiji Ministry of Health has adopted a multi-sectoral approach to combat NCDs and the associated risk factors like obesity, in line with global recommendations. National policies whose immediate focus is not health can impact on population health; health gains can be achieved when national policies outside the health sector are influenced [5]. Improving dietary patterns remains a particular challenge for the Pacific Islands, with an obesogenic food environment [6] now being commonplace. While a number of innovative food policy initiatives have been implemented [6], more are needed and the effectiveness of any new policy needs close monitoring.

Palm oil is a vegetable oil sourced from the oil palm tree native to West Africa [7] and is categorised under vegetable oils in the Fiji Food Safety Regulation. It is used in a wide variety of food products like cooking oil, shortening and margarine. It is high in saturated fat and low in polysaturated fat and may contribute to obesity [8] and heart disease [9]. When partially or fully hydrogenated, palm oil induces an adverse effect on plasma lipid profile, free fatty acids and phospholipids which are major contributing factors to obesity [10]. Hydrogenation is a process used to enhance the shelf life of food products. Food manufacturers often prefer palm oil over other cooking oil due to its heating ability and low market price. Consequently, processed palm oil is widely distributed and it dominates the global cooking oil market with over 60% share in the 2015 and 2016 trading period [11].

Given the atherogenic lipid profile of palm oil, countries are increasingly exploring strategies to reduce consumption. Some developing countries have intervened to control market prices of palm oil, but the impact of these initiatives on consumers is unknown. For example, the Ministry of Health in East Timor were looking at the adoption of national policies that limits the use of modified vegetable (palm) oil [12]. The government of Thailand carried out a study to fully understand the prospects for future health-focused policy development to limit food use of palm oil [13].

In the 2012 budget, Fiji announced an increase in the fiscal duty on imported palm oil from 15 to 32% with the duty applied once the product reached the port of entry. The aim of the duty change was to increase the price of the product, reduce consumer purchasing and lower population saturated fat intake. This policy change was made in response to a submission received by the budget sub-committee from the Fiji’s Ministry of Health, which included recommendations for tax adjustments on healthy and less healthy foods, regulation of food marketing, and improving accessibility of healthy food in schools and promotion of local foods.

This study aims to document the process that led to the implementation of the 2012 palm oil duty and to assess its impact. In particular we wanted to obtain policy maker and stakeholder perspectives on facilitators that assisted or barriers that slowed adoption, factors influencing implementation and to track the impact of the duty on consumption using import volumes.

## Methods

### Study design

A qualitative approach based on case study methodology was used to investigate the introduction of the new import duty on palm oil in Fiji. This included a 2014 desk-top assessment of publically available documents related to the policy change, including media reports, public documents, reports, newsletters and organisational websites. Documents published since 2010 were identified by a tailored search of Fiji government (sites ending in gov.fj) for documents published in English. We defined Government policy documents’ as those government institutions and organisations can be held accountable for and searched these using ‘palm oil’ as the key words. We also manually searched Fiji media websites (e.g. fijitimes.com) for ‘palm oil’ using the Google search tool. Two auditors screened and reviewed the results of each search strategy to determine which records were eligible for inclusion. Secondly, the assessment of impact of the import duty was based on analysis of existing datasets and semi-structured interviews with key informants from government and non-government organisations between 2015 and 2016. Barriers and facilitators to policy change were also assessed through the interviews.

### Recruitment

Potential participating organizations were those previously involved in the Pacific Obesity Prevention in Communities (OPIC) [14] project as well as the Translational Research on Obesity Prevention in Communities (TROPIC) [15] program in Fiji. Participants were selected based on their involvement with the policy submission. Endorsement for the research team to work with selected government ministries was secured from their respective Permanent Secretary or General Manager/Chief Executive Officer of other stakeholders. Letters of invitation plus a Personal Information Sheet and consent forms were then mailed to individual participants. Based on documentation of the implementation process and our awareness of who was involved, we anticipated conducting at least 10 semi- structured interviews.

### Policy context

Whilst policy development processes vary, a typical pathway for developing fiscal policy change in Fiji is outlined here. In advance of the budget development process, invitations are sent to various stakeholders, government ministries and quasi-governmental organizations requesting submissions for fiscal changes. A budget sub-committee consisting of officials from the Ministry of Finance and Fiji Revenue and Customs Authority is assigned the task of scrutinising all submissions for feasibility and environmental impact. After a thorough review of submissions, the budget sub-committee develops a short list of proposals deemed feasible to submit to Cabinet for final review, approval and endorsement. Once approved, the fiscal changes await the annual budget address for announcement.

### Data collection

**Fig. 1:**
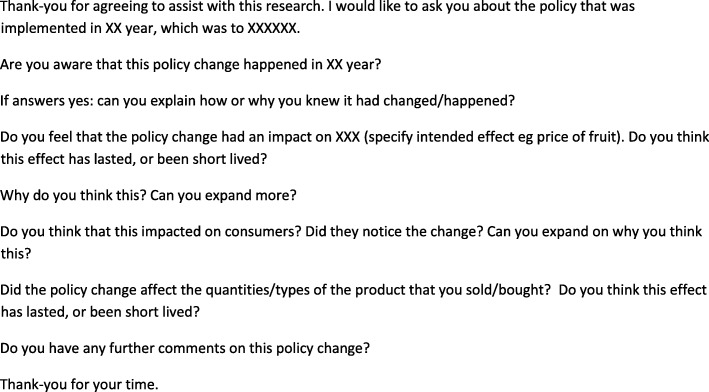
Semi structured questionnaires A2 showing questions assessing perceived impacts of policy changes with selected store owners, importers and staff from the Ministry of Health; and A3 assessing drivers, facilitators and barriers to policy change with selected participants from selected government ministries and civil society Groups

Semi-structured interviews (Fig. 1a, b) of approximately 30 min’ duration were conducted in English from August 2015 to February 2016, generally in the interviewee’s office at a time convenient to them. Palm oil import data were obtained from the Fiji Bureau of Statistics for the years 2010 to 2015 to ascertain trends in import volumes (tonnes/year); these were verified in interviews with customs officers.

### Data management and analysis

Interviews were audio recorded, and transcribed. The identities of participants were coded by private sector or government, but were de-identified. Transcripts were validated by a second member of the research team. Simple thematic analysis was undertaken to identify perceptions on impact and issues/strengths with implementation drawing on the guidance on the conduct of narrative synthesis of Popay et al. [16] Using Walt and Gilson policy triangle 354 [17, 18] as a framework, participant responses were categorised into themes. Participant confidentiality was maintained by using unique identifiers to code transcripts.

Information gathered from the desktop assessment was summarised in a matrix to facilitate comparison with other food policy recommendations. The matrix was designed to track the progress of all policy recommendations from OPIC (i.e. what has happened to each of the policies and in what stage of implementation have these policies reached as of 2016?).

## Results

### Participants

Fourteen participants completed the semi-structured interviews. Participants represented the Consumers Council of Fiji (*n* = 2), the Ministry of Health (*n* = 3), Commerce Commission (*n* = 2), National and Food Nutrition Centre (*n* = 2), Fiji Revenue and Customs Authority (2) and supermarkets/ importers (*n* = 3). The overall response rate was 82% (14/17). Two potential participants could not be contacted; one had retired and the other had changed offices.

### Desktop assessment

Apart from 2012 budget announcement by the Prime Minister, there were few local media documents that directly captured the implementation or impact of the import duty. Twelve relevant documents were found in the desk-top review. The documents revealed that a tenfold increase in palm oil imports into Fiji between 2000 to 2009 had triggered concerns about its use in homes and the local food industry [6]. In 2010, Fiji’s Ministry of Health conducted a desk-top comparative analysis of all cooking oils used locally. They found that palm oil was the least healthy oil consumed compared to other varieties available like canola and pure virgin coconut oil. This analysis led to calls by the Ministry of Health for consumers to opt for a more healthy oil than palm oil [19].

There is no palm oil production in Fiji, with the country reliant on imports from Indonesia, Singapore and, in particular, Malaysia [20]. In 2011, the Ministry of Industry and Trade held talks with Malaysian investors on the prospect of setting up a palm oil industry in Fiji, however we found no evidence of the proposed collaboration eventuating [21]. Fiji’s Prime Minister, Commodore Bainimarama during the 2012 budget address declared, “*As a part of the overall strategy, we will increase the tariffs on the importation of palm oil and monosodium glutamate (MSG)—both of which negatively affect health. They will now have a duty rate of 32 percent”* [22]. This apparent change in official attitudes towards palm oil represented a significant turn of events. A palm oil industry remains a possibility however as a Malaysia-Fiji business council was launched in 2014 to encourage more trade and investment between the two countries [23].

Following implementation, a Consumer Council budget submission (2013) highlighted the need for government to review the increase in palm oil duty. This call stemmed from surveys conducted by the Consumer Council that revealed importers, retailers and wholesalers were re-labelling palm oil as vegetable oil [24]. This study assessed this issue, and identified that labelling palm oil as vegetable oil was not prevented under the Fiji Food Safety Regulation [25] and, therefore labelling a product as vegetable oil when its palm oil was not contravening these regulations. However, declaring palm oil as vegetable oil on an important manifesto for customs purposes would be contravening the local customs law.

The Pacific Economic Monthly Report (December, 2015) suggested that palm oil imports were amongst products doing poorly in the market [26]. Pacific Island countries trade with each other and there are agreements (like the Pacific Island Countries Trade Agreement (PICTA) and the Melanesian Spearhead Group or MSG) to facilitate trade between members [27]. One of the underlying factors in trade agreements is the removal of trade barriers among trade partners [6]. In the Pacific Islands, only Papua New Guinea (PNG) grows palm oil but there was only one importation of palm oil from PNG to Fiji in 2013 [20].

### Participants’ perspective on facilitators to policy implementation

Most interviewees were well aware of the palm oil tax submission process. Widespread public support and inter-sectoral commitment coupled with a comprehensive cabinet policy briefing paper and firm leadership were prominent among the responses from most participants as factors facilitating the policy’s implementation. Also, according to interviews with customs officers, the new import was instantly implemented on budget announcement day (25 Nov, 2011) since it is a perishable good.

Awareness of the detrimental health impact of palm oil was identified as a facilitator by participants within and outside of the health sector.

*“… and then of course, it is associated with a lot more saturated fat and then you know… so I was concerned about the health of our people,* ***so I said no*** (no to palm oil)*. The only way of doing it was to raise the tax to 32%”*. (S5003).

One participant went further and identified pricing as a strategy for protecting health.

*“We don’t want it to be cheap because if you make it cheap, people will buy it. So, the intention was to make it more expensive to deter consumers from actually purchasing it”.* (Q5005).

Availability of evidence was also a facilitator.

*“… What I like about this policy is that it’s evidence base. You know from OPIC we shifted to TROPIC and this is really a new area for Fiji.”* (S5002).

Another facilitator was inter-sectoral support from key players within government ministries at key stages. Several quotes pointed to good inter-sectoral support and collaboration.

*“But as I* (S5002) *say it’s the external support that drove it… and push it through… in my opinion”.*

*“I think what really brought it to the fore is because of support, strong vocal support from* xxx*”* (Q5001).

*“It went smoothly because the understanding was that there was a good consultation with the health ministry.”* (S5006).

The presentation of a clear and comprehensive submission which highlighted evidence of the health impacts of palm oil and the timing of the submission was a third facilitator. The submission was prepared by a Ministry of Health officer and most participants agreed that the thoroughness of the submission was a significant driver towards its successful implementation.

*“I think our submission to parliament was clear, they were quite clear from the policy briefs.”* (S5009).

One participant pointed out the use of external evidence in the submission.

*“I think for that one* (Palm Oil) *the objective was clear. There were reports coming in from XXX of the risks that palm oil imposes on the health of Fijians, so it was part of the submission that came in.”* (S5013).

While participants considered both the timing and completeness as important, one singled out timing of the submission as being of most importance.

*“I think it was the combination of timing, the right time… the government at that time being interested in supporting the Ministry of Health with taxation changes and then having well documented briefs* (Palm oil policy brief) *particularly the one on palm oil which is very comprehensive.”* (P5012).

The simplicity of the submission was also appealed.

*“So, I presented all of those and we discuss them and the Minister was very keen of the fact that they have already written up a policy brief. He was particularly keen on the palm oil one as oppose to the other more complicated duty change that were proposed to alter the intake of the unhealthy oils”.* (P5012).

A final facilitator was drive and decisive leadership from senior government figures and key decision makers.

*“Not to mention the Minister (laughs)… Minister for health back then. Because he was always on the… you know… yeah when he was always on the beat he’d say “get it done”…and you have to get it done or you are done (laughs)”* (Q5001).

Certain organisations were seen as instrumental to the policy’s implementation and even individuals were hailed following the success of the palm oil policy submission.

*“I think the Minister tends to rely on the technical support from XXX….”* (S5008).

### Barriers to policy implementation

The only obstacle identified to implementation included counter lobbying from traders, retailers and importers.

*“So, this issue with palm oil – they* (Food Industry*) are against it. So, they will write to the Minister - they have every right”* (S5002).

*“In my recollection of what’s going on, it’s a fact that there might be some objections from the restaurants. And I think there might be some objections from the food industry because the food industry also uses that* (palm oil) *as an ingredient in some of their products like biscuit and stuff like that, because they* (palm oil) *are cheaper”* (Q5001).

One participant gave an insight into how local traders were trying to oppose the new palm oil duty change,

*“I think what happens… the companies, the trade companies XX and group was you know… trying to push that we don’t do that because they were bringing it in but they did, in that point in time we were able to push through without too much….* (Opposition)*.”* (S5003).

### Unanticipated barriers to policy effectiveness

Some participants identified the labelling of palm oil as vegetable oil, which occurred after the tax increase in 2012, as a factor that may undermine the impact of the tax and potentially mislead consumers.

*“We have yet to really determine the impact, we are working with the palm oil part… but we have a problem with palm oil. They have changed, they’ve removed the palm oil and uses vegetable oil as the* (label)*… which is according to our food safety act, and that’s the problem with palm oil”.* (S5002).

*“I think the imports reduced, but we just found out that palm oil is still there, they just change the… to vegetable oil, without mentioning palm. So, the trickle-down effect is that it really effect the importing practice. Now they know that we are coming after palm oil, they’ve change… so the trickle-down effect in that sense is that it has made traders more notorious… now they playing around, and palm oil in the food safety act* (regulation) *is vegetable oil, so ---we need to re-look at that policy. In the Food Safety Act they just say vegetable oil and palm is considered as a vegetable”*. (Q5005).

Comment was also made about consumers’ lack of understanding about palm oil being sold as vegetable oil and the need for education campaigns.

*“I think one of the issues is that very few people understood the risks of palm oil as vegetable oil”*. (Q5001).

*“You know vegetable oil is being promoted as the healthier option. So yeah, suppose the Ministry of Health and particularly the Nutrition Centre hadn’t actually done its homework* (Awareness campaigns) *in my opinion in terms of advising the public on the differences about these vegetable oils”.*

### Participants’ perceptions of impact

Participants described impacts on price and availability. With respect to price, participants described increases but also reasons why increases may not be noticeable.

*“Yes… yes the price* (of palm oil) *has increased”* (R5010).

*“We have removed certain products out of price control, those products with health related issues. So, corn mutton, corn beef, vegetable palm oil has been removed…what I’m trying to say is once the price control is removed then there’s no restrictions on the margin you can place. So, what the traders can do then is put the margin that they desire, and this often results in price increase, which discourages consumption”.* (S5004).

The Price Control List (PCL) includes all names of basic food products that are restricted within a price range. Palm oil’s exclusion from the updated PCL means traders can alter or increase prices of palm oil as they wish.

Some noted that palm oil was missing from supermarket shelves since the policy change and that purchasing behaviour had changed.

*“For palm oil not necessarily the price impact but what we’ve noticed from our surveys - our recent surveys is that there’s less of it on the shelves. If you go to XXX supermarket, which is the closest supermarket here, I don’t think you will find palm oil there”.* (Q5005).

*“Yes, it has an impact a lot, by doing that people have switched from palm oil to other better option like soya bean, canola and some coconut oil. Some flora canola is known best for the heart and actually you won’t see any major retailers selling palm oil in there store. For us we have stopped selling from last two years”.* (R5012).

*“No, we are not concentrating too much on prices. Because right now we can’t find any so there’s no use talking about price…*” *.* (S5003).

Another noted that the reduction in palm oil availability may have been due to the labelling.

*“Yeah palm oil has decreased except for that changing in the names. We just have to verify has it really decreased or they just changing the names”.* (S5002).

## Discussion

This study analysed documentation and presented perspectives of selected key informants on the process of implementing a new palm oil import duty in Fiji. Our study revealed that actors included the Ministry of Health, consumer council, Ministry of Finance and the customs authority. The context for implementation was a health sector drive to reduce non-communicable disease by taxing unhealthy foods (palm oil) and the content of the policy was an increase import duty on palm oil (from 15 to 32% of the purchase price) designed to raise shelf prices and discourage importation from traders [18]. The implementation process appeared to be straight forward and we found some evidence of a reduction in imports of palm oil after the duty was applied.

### Impact of the import duty on volumes

The study shows that imports of palm oil were dropping from 2011 but also a significant drop in the volume of palm oil imports when the tax policy came into effect in 2012. The volume declined from a peak of over 5000 t in 2011 to just over 2000 t in 2015. Since then, the volume of imports has continued at a lower level.

#### Implementation of the palm oil duty

The palm oil submission advanced seamlessly through a typical policy process despite some initial resistance from the food industry. Loopholes in the existing regulation were identified that allowed retailers to re-brand palm oil as vegetable oil potentially misleading consumers trying to avoid palm oil. This was summed up by a participant.

*“… so we are really talking about 10 to 15 years before the impact reflects on the consumers which is where I really wanted this to reach….I think it will take time”.* (S5002).

Key facilitating factors were awareness, inter-sectoral support, a comprehensive policy brief submission, and strong leadership. Most actors were aware of the ill-effects of palm oil on health. They were also aware of other actors’ behaviour and the ongoing collaboration between government institutions and quasi-organizations in trying to increase the palm oil import duty. Previous work highlighting the association between palm oil and NCDs, captured on the comprehensive policy brief, may have contributed to this. Good leadership shown by certain individuals was also hailed as a facilitating factor, as was the simplicity of the policy change when compared to other complex tax changes. These findings are consistent with other studies [28, 29] that show leadership, collaboration of important partners and the awareness or understanding of the issues by key figures as facilitating factors in health policy implementation.

Evidence and advocacy from health researchers [29] plays a critical role in shaping the demand for healthy oils [30] and this proved to be the case in for the palm oil duty with the policy brief creating an opening for informed dialogue with relevant ministries [15]. Collaboration was forthcoming in this instance due to the early engagement of key partners during the policy formulation stage and the policy brief brought an understanding of the issue to sectors outside health that had a say in the process. More cynically, some participants suspected that the swift adoption of the duty may have been driven by the potential revenue flow. Some deemed that it favoured the political agenda of the day so explaining its swift adoption.

Barriers to implementation were minimal. The increase in the palm oil import tax was announced by the Prime Minister in 2012 which was before the national general election in 2014. Counter lobbying from the food industry was noted but appeared to be piecemeal or not clearly documented regarding the palm oil submission. Also, palm oils market share may be small enough that industry did not see value in mounting strong opposition.

#### Impact of the duty

In our study, participants described increases in the price of palm oil. Other countries have made similar efforts to limit the consumption of palm oil. In 1987, the Mauritian government changed cooking oil from mostly palm oil to wholly soya bean oil to protect the health of the population [31], a survey on the population’s fatty acid composition was also done to serve as baseline data. The consequent survey in 1992 found that the total cholesterol concentration fell significantly; this was attributed to the switch to soya bean oil. The Mauritius study illustrates the time needed to demonstrate the impact of public policy interventions on health. Another study modelled the impact of a hypothetical tax change on the Indian population [32] to predict future mortality. A 20% tax on palm oil was expected to avert 363,000 deaths from myocardial infarction and strokes over the period 2014–23 in India. In 2016, the French government dropped a proposed palm oil tax; the French media was quick to speculate that the country was forced to drop the palm oil tax due to threat of economic retaliation from Indonesia [33].

In Fiji, taxation of palm oil is done at port of entry, so labelling of palm oil as vegetable oil on packaging will not help importers avoid the import duty. However, it may partially protect palm oil sales (Additional file 1) in stores by misleading consumers into thinking they are purchasing a different type of vegetable oil and the general trend of declining import volumes coinciding with the tax implementation could be cited as possible support for the tax’s effectiveness. For example, if the front of pack label says ‘vegetable oil’, the only way consumers can determine which type is by reading the ingredient label. While relabelling of palm oil as vegetable oil is lawful since it is categorised under vegetable oil in the Food Safety Regulations, advocacy is needed to help consumers be more aware of what they are purchasing. There was some media coverage in 2013 when Fiji’s Consumer Council raised this issue, and it is possible this contributed to reduced sales and import volumes.

The main strength of this study was first hand, rich information drawn from personnel within and outside government who were directly involved in the palm oil submission. A short survey in supermarkets to confirm the availability or re-labelling of palm oil could further provide stronger arguments on the accessibility and rebranding of palm oil in Fiji. There were a number of limitations. We had a convenience sample of policy-makers, government officers, private organisations CEO, and they may not have captured all the barriers and facilitators. The unique political environment in Fiji at the time of implementation may make it hard to generalise the implementation process to other settings. Although import volumes showed a decrease, we cannot directly attribute declines to the increased import duty. We also cannot directly determine if the increased duty affected price or consumption. Further studies on retail prices and consumer consumption level are needed, including drawing information from the recently completed National Nutrition Survey.

## Conclusion

The successful introduction of an increased palm oil import duty was credited to the drive and consistent lobbying from public health fraternity. Other factors like a thorough policy draft and clear evidence on the ill effects of palm oil on health were also identified as major catalysts for the change. It was suspected by some participants that retailers and importers make use of the gaps in the existing food regulations to confuse consumers into believing they were buying healthier oils.

Some recommendations for future area of actions could include a store survey to determine how many cooking oil brands contain palm oil. A study on palm oil purchasing pattern by food processors and a survey on the quantity of palm oil used in food products will facilitate mapping of the amount of palm oil available at a population level. Analysis of successive national nutrition surveys and NCD surveys may reveal trends in vegetable oil consumption.

## Additional file

Additional file 1: Palm Oil Trend in Fiji from 2010 to 2015. (DOCX 15 kb)

## Abbreviations

MSG: Melanesian Spearhead Group
NCD: Non Communicable disease
OPIC: Pacific Obesity Prevention in Communities
PCL: Price Control List
PICTA: Pacific Islands Countries Trade Agreement
TROPIC: Translational Research on Obesity Prevention in Communities
WHO: World Health Organization
