# Supplementary material for: The development and implementation of a new import duty on palm oil to reduce non-communicable disease in Fiji
Source: Global Health. 2018 Aug 29;14:91. doi: 10.1186/s12992-018-0407-0 (PMC6116374; doi:10.1186/s12992-018-0407-0)
Supplement: Supplementary file 1 — Palm Oil Trend in Fiji from 2010 to 2015. (DOCX 15 kb) [file 12992_2018_407_MOESM1_ESM.docx]

Palm oil import trend in Fiji, the dotted line indicates when the duty change was imposed

Source: Fiji Bureau of Statistics
